# Serogroup C *Neisseria meningitidis* disease epidemiology, seroprevalence, vaccine effectiveness and waning immunity, England, 1998/99 to 2015/16

**DOI:** 10.2807/1560-7917.ES.2019.24.1.1700818

**Published:** 2019-01-03

**Authors:** Helen Findlow, Helen Campbell, Jay Lucidarme, Nick Andrews, Ezra Linley, Shamez Ladhani, Ray Borrow

**Affiliations:** 1 Vaccine Evaluation Unit, Public Health England, Manchester Royal Infirmary, Manchester, United Kingdom; 2 Immunisation Department, Public Health England, Colindale, London, United Kingdom; 3 Meningococcal Reference Unit, Public Health England, Manchester Royal Infirmary, Manchester, United Kingdom; 4 Statistics, Modelling, and Economics Department, Public Health England, Colindale, London, United Kingdom; 5 University of Manchester, Infection, Immunity & Respiratory Medicine, School of Biological Sciences, Stopford Building, Manchester, United Kingdom

**Keywords:** bacterial meningitis, epidemiology, Neisseria meningitidis, surveillance, vaccines, immunisation

## Abstract

**Background:**

In 1999, the United Kingdom (UK) was the first country to introduce meningococcal group C (MenC) conjugate vaccination. This vaccination programme has evolved with further understanding, new vaccines and changing disease epidemiology.

**Aim:**

To characterise MenC disease and population protection against MenC disease in England.

**Methods:**

Between 1998/99–2015/16, surveillance data from England for laboratory-confirmed MenC cases were collated; using the screening method, we updated vaccine effectiveness (VE) estimates. Typing data and genomes were obtained from the Meningitis Research Foundation Meningococcus Genome Library and PubMLST *Neisseria* database. Phylogenetic network analysis of MenC cc11 isolates was undertaken. We compared bactericidal antibody assay results using anonymised sera from 2014 to similar data from 1996–1999, 2000–2004 and 2009.

**Results:**

MenC cases fell from 883 in 1998/99 (1.81/100,000 population) to 42 cases (0.08/100,000 population) in 2015/16. Lower VE over time since vaccination was observed after infant immunisation (p = 0.009) and a single dose at 1–4 years (p = 0.03). After vaccination at 5–18 years, high VE was sustained for ≥ 8 years; 95.0% (95% CI: 76.0– 99.5%). Only 25% (75/299) children aged 1–14 years were seroprotected against MenC disease in 2014. Recent case isolates mostly represented two cc11 strains.

**Conclusion:**

High quality surveillance has furthered understanding of MenC vaccines and improved schedules, maximising population benefit. The UK programme provides high direct and indirect protection despite low levels of seroprotection in some age groups. High-resolution characterisation supports ongoing surveillance of distinct MenC cc11 lineages.

## Introduction

*Neisseria meningitidis* is a major cause of meningitis and septicaemia worldwide. Efforts to control meningococcal disease have been aimed at the development of effective vaccines and subsequent implementation in appropriate immunisation schedules. Twelve different meningococcal serogroups are recognised and serogroup B (MenB) is currently responsible for most cases of invasive meningococcal disease (IMD) in Europe. Many countries, including the United Kingdom (UK), experienced large outbreaks of serogroup C (MenC) disease in the mid-1990s, due mainly to the ST-11 clonal complex (cc11) [1]. In 1999, the UK became the first country to introduce the MenC conjugate (MCC) vaccine in a phased national campaign targeting all those aged less than 18 years over a 12-month period, alongside a routine three-dose infant programme [2]. Vaccine eligibility was later extended up to 24 years of age, although this age group was not actively called for vaccination. Routine use of MCC vaccine in many other European countries followed [3].

As MCC vaccines were licensed on immunogenicity studies alone, without direct evidence of clinical efficacy, comprehensive national surveillance was initiated concurrently in order to monitor the vaccines’ impact in a population-based setting [2]. MCC vaccination was associated with rapid and sustained declines in MenC disease across all age groups through direct and indirect (herd/population) protection [3-5]. Invasive disease is rare following nasopharyngeal acquisition where the meningococcus can persist for several months before it is cleared; this asymptomatic carriage, especially in older teenagers, is an important reservoir for infection, onward transmission to susceptible individuals and immunity. In the UK, there was a 66% reduction in MenC carriage among 15–19 year-olds within 1 year of MCC vaccine implementation and this reduction was key to establishing indirect protection across the population [6].

The UK MCC immunisation programme has evolved over time (Box) and, from 2013, has included an adolescent MCC vaccine programme to extend direct protection in teenagers and maintain indirect protection in the wider population. In 2015, this vaccine was replaced with the quadrivalent meningococcal conjugate vaccine (MenACWY) to combat a national MenW outbreak. A multi-component, protein-based vaccine against MenB was also implemented in the infant immunisation schedule at the same time [7]. In July 2016, the infant MCC dose was removed because MenC cases were extremely rare in this age group and population protection was likely to be maintained through the adolescent MenACWY programme.

Box **Summary of the changing United Kingdom meningococcal immunisation schedule, 1999–2016**

1999: Routine infant vaccination at 2, 3 and 4 months of age MenC vaccine

- Catch-up of children aged 5 months to 18 years MenC vaccine

2006: Routine infant vaccination at 3 and 4 months of age MenC vaccine

- Booster (of MenC) at 12 months of age MenC/Hib vaccine

2013: Routine infant vaccination at 3 months of age MenC vaccine

- Booster (of MenC) at 12 months of age MenC/Hib vaccine

- Booster at 13–15 years of age MenC vaccine

2015: Routine infant immunisation at 2 and 4 months of age MenB vaccine

- Routine infant immunisation at 3 months of age MenC vaccine

- Booster at 12 months of age MenB vaccine

- Booster (of MenC) at 12 months of age MenC/Hib vaccine

- Routine teenage dose at 13–15 years of age MenACWY vaccine

- Catch-up of teenagers aged 15–18 years MenACWY vaccine^a^

2016: Routine infant immunisation at 2 and 4 months of age MenB vaccine

- Booster at 12 months of age MenB vaccine

- Primary dose (of MenC) at 12 months of age MenC/Hib vaccine

- Routine teenage dose at 13–15 years of age MenACWY vaccine

^a^ Completed in 2017.

Given resource limitations, it is important that vaccination programmes are optimally delivered to ensure maximal population benefit. Here, we evaluate the long-term impact of a national MCC vaccination programme in England on (i) MenC epidemiology, (ii) the clinical characteristics of confirmed MenC cases, (iii) MCC vaccine effectiveness (VE), (iv) characterisation of MenC isolates from invasive cases, and (v) population immunity through a national serosurvey that updates previous studies [8-10].

## Methods

### National epidemiology

Public Health England (PHE) conducts enhanced national surveillance of IMD in England. The PHE Meningococcal Reference Unit (MRU) provides a national service for confirming, grouping and characterising invasive meningococcal isolates [11]. The MRU also provides free national Polymerase Chain Reaction (PCR)-testing of clinical samples from patients with suspected IMD; Diagnostic laboratories are required by law to notify PHE when they identify *Neisseria meningitidis* and samples are requested to be sent to the MRU for confirmation and characterisation. PHE routinely reconciles laboratory-confirmed cases to generate a national dataset [12] and follows up each case for demographic data, vaccination history, clinical presentation and outcomes through Health Protection Teams (HPTs), general practitioners and, for younger cases, hospital clinicians. Details documented on HPZone (a web-based software for public health management of infectious diseases used by HPTs throughout England), were also accessed by PHE to obtain additional information on cases since 2010.

### Vaccine effectiveness

Using the national dataset, MCC VE was calculated using the screening method [13] for confirmed MenC cases among MCC-eligible individuals (born since 1 September 1981) in England between 01 January 2000 and 30 June 2016. VE was estimated according to time since vaccination (or, if unvaccinated, since the age at last scheduled dose); analysis included unvaccinated children, those who had completed the recommended primary immunisation course for their birth cohort (three doses for those aged 1 year of less before September 2006), or had received one dose after their first birthday at any age. Effectiveness of the primary plus booster dose was based on cases who had received ≥ 1 primary MCC dose followed by the 12-month Hib/MCC booster. The following formula was used:

VE = 1 − [PCV(1 − PPV)]/[(1 − PCV)PPV]

Where PCV is the proportion of vaccinated MenC cases and PPV is the proportion of vaccinated population (i.e. vaccine coverage, matched to each case based on their age and birth cohort and then averaged). Annual national vaccine coverage by 12 months of age (from April 2001) and by 24 months of age (from April 2002) was used for the latter [14].

Data extracted in May 2016 from The Health Improvement Network (THIN) database [15] for children born from August 1999 to May 2015 was used to estimate partial vaccination proportions for each of the routine schedules (3 + 0, 2 + 1, 1 + 1) by 12 months and 24 months of age. These estimates of partial vaccination were used to adjust the national coverage estimates to give coverage in those who received either no vaccination or the full the schedule. For example, it was estimated that in the three-dose primary cohort, 5.5% received one or two doses by 12 months, so national coverage of 90% for three doses would be adjusted to (90*100)/(100–5.5) = 95.2%, once partial vaccination is excluded.

### Molecular characterisation

Typing data and genomes were obtained from the PubMLST *Neisseria* database and Meningitis Research Foundation (MRF) Meningococcus Genome Library (MGL), to which all MRU isolates from the national dataset have been referred since July 2010 [16,17]. A recent phylogenetic network analysis of the known population structure of MenC-associated cc11 sublineages was re-annotated in the context of the current study since cc11 represented the majority of recent invasive MenC isolates in England [18]. Briefly, all available global cc11 genomes, including 121/122 invasive MenC isolates from England (2010/11–2015/16), and excluding serogroup W-associated lineage 11.1 sublineages (final n = 898 globally; accessed 7 October 16), and two non-cc11 reference genomes (IDs 27778 and 29645; ccs 41/44 and 8, respectively), underwent core genome comparisons (1546 loci) using the PubMLST genome comparator tool. The resulting distance matrices were visualised using SplitsTree4. The invasive MenC cc11 isolates collected in England during 2010/11–2015/16 (inclusive) were highlighted [19].

### Serosurvey

Serum samples collected in 2014 were obtained from the PHE Seroepidemiology Unit; a depository of anonymised residual sera from routine diagnostic testing at participating laboratories. Known immunocompromised individuals are excluded from the collection. The age, sex and year of collection were known for individual samples: immunisation status was not known [20].

Serum bactericidal antibody (SBA) assays were performed against the serogroup C target strain, C11 (phenotype C:16: P1.7–1,1) as previously described [21]. The complement source used in the SBA was pooled serum from 3–4 week old rabbits (Pel Freez Biologicals, Arkansas, United States (US)). Titres were expressed as the reciprocal serum dilutions yielding ≥ 50% killing after 60 minutes. The lower limit of detection was a titre of 4. Titres of < 4 were assigned a value of two for geometric mean titre (GMT) analysis. Titres of ≥ 8 were considered protective against MenC disease [22].

Approximately 100 samples were selected from each age-band to fit with different vaccine schedules since MCC introduction and to allow comparison with previous studies. This sample size was selected to achieve reasonable precision around the 95% confidence intervals (CIs) for proportions with SBA titres ≥ 8 and GMTs within the age group of interest.

Since MCC vaccine coverage has been consistently high, the age of the serum donor was used to align the donor with the MenC vaccine schedule they should have been offered and thereby identify which MenC vaccine cohort they belonged to. This then allowed comparison of protection between schedules and over time, using historical data from three previously published similarly designed surveys. These earlier surveys used sera collected from the same Seroepidemiology Unit and tested using the same methodology in the same PHE laboratory during 1996–1999 [8], 2000–2004 [9] and 2009 [10].

## Results

### Epidemiology

**Figure 1:**
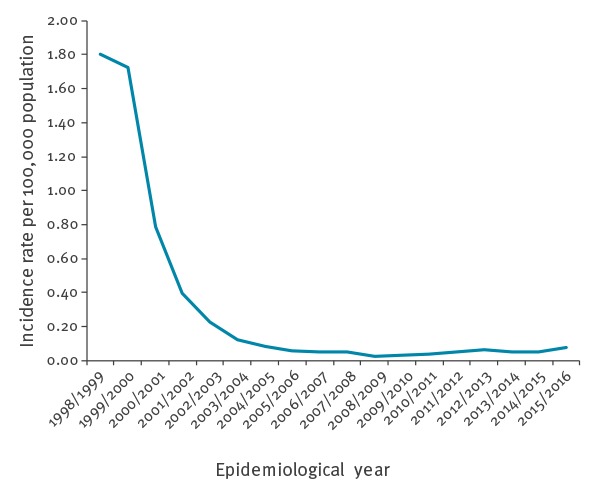
Incidence rate of group C meningococcal disease by epidemiological year, England, 1998/99–2015/16

MenC cases in England fell rapidly from 883 in 1998/99 (pre-vaccination year, incidence 1.81/100,000) to 13 cases in 2008/09, continuing at low levels (17–33 cases annually) during 2009/10–2014/15. In 2015/16, there were 42 cases (0.08/100,000), similar to disease levels last observed in 2004/05 (Table 1, Figure 1). Over the last decade, the age distribution has been relatively constant (Figure 1). However, the proportion of cases among those aged 5–14 years increased from 12% between 2006/07–2010/11 (0.04/100,000) to 17% between 2011/12–2015/16 (0.09/100,000), while the proportion of cases among 1–4 year-olds declined from 11% (0.09/100,000) to 4% (0.05/100,000) over the same period. Cases in those aged 45 years and over (who were not vaccine eligible) were 86% lower in 2015/16 (n = 16) than 1998/99 (n = 111) consistent with a sustained population protection effect.

**Table 1:** Total number of confirmed group C meningococcal disease cases, England, 2006/17–2015/16 (N = 268)

Epidemiological year	Age group (years)									
	< 1	1-4	5–9	10–14	15–19	20–24	25–44	45–64	≥ 65	Total
2006/07	2	2	2	0	1	3	7	7	4	**28**
2007/08	1	2	1	0	4	2	7	4	7	**28**
2008/09	0	1	1	2	0	0	1	7	1	**13**
2009/10	1	4	3	1	1	0	5	2	0	**17**
2010/11	2	3	2	1	1	0	6	4	3	**22**
2011/12	3	3	2	1	3	2	8	4	3	**29**
2012/13	0	1	1	4	3	3	9	7	5	**33**
2013/14	1	2	5	2	0	1	7	6	3	**27**
2014/15	1	0	6	0	3	0	10	7	2	**29**
2015/16	1	1	4	3	3	1	13	7	9	**42**
**Total**	**12**	**19**	**27**	**14**	**19**	**12**	**73**	**55**	**37**	**268**

Distribution of laboratory-confirmed cases of group C meningococcal disease by age group over the most recent 10 epidemiological years, England, 2006/07–2015/16.

Between 2011/12 and 2015/16, 160 MenC cases were confirmed and 18 died (case fatality rate (CFR), 11%). The London region, where 16% of the English population resides (8.7 of 54.8 million) accounted for 41% of cases (n = 66). Overall, 82 cases were female and 78 male but the sex distribution differed by age with a preponderance of males aged 25–44 years (30/47, 64%) and of older female cases aged ≥ 45 years (35/53,66%). Thirty-two cases (20%) had a history of recent travel, 17 cases were born outside the UK and were more likely to have recently travelled (15/17, 88%) than individuals born in the UK (17/143, 12%).

Of the 160 cases, 11 (7%) were living in shared accommodation (three university students, four in care homes or assisted living, four in house share or hostel accommodation). There were six cases in known men who have sex with men (MSM) and eight individuals reported using recreational drugs, were injecting drug users or had alcohol dependency. Nineteen cases (12%) had underlying co-morbidities; heart condition (n = 3), diabetes (n = 2), HIV (n = 6), immunosuppression (two on psoriasis treatment, one with chronic lymphocytic leukaemia, one post liver transplant), chronic respiratory condition (n = 3), liver disease (n = 3). There were three clusters of two cases each in primary schools nationally in 2015; all six children were fully-vaccinated (two-dose primary course with booster).

### Vaccine effectiveness

Between 1 January 2000 and 30 June 2016, there were 316 MenC cases in vaccine-eligible individuals, of whom 119 had been fully-vaccinated according to their recommended schedule (Table 2). Estimated VE within 12 months of immunisation at all targeted ages (including infants) was high at 91% to 96%. Following routine infant vaccination or a single dose administered to cohorts aged 1 to 4 years when the catch-up programme began, a trend for lower VE with increasing time since vaccination was observed (p = 0.009 and p = 0.03 respectively) (Table 2). In school-aged cohorts (aged 5–18 years when immunised), high VE was sustained over time, remaining at 95.0% (95% CI: 76.0 to 99.5%) at least 8 years after vaccination. Of the 26 cases eligible for the Hib/MenC booster, 25 were vaccinated, with matched coverage of 94.6%; VE was -43% but with wide confidence intervals (95% CI: -5,759 to 77). This calculation is very sensitive to the number of unvaccinated cases; for example, the addition of just two unvaccinated cases would change this estimate to 52%.

**Table 2:** Meningococcal serogroup C conjugate vaccine effectiveness based on the screening method in immunised cohorts, England, September 2000–June 2016

Cohort	Approx age at MCC vaccine	Average matched population coverage %	Proportion of cases vaccinated^a^ n/N	Proportion of cases vaccinated %	Overall vaccine effectiveness % (95% CI)	Vaccine effectiveness based on time since vaccination % (95% CI)		Trend analysis^b^
						Within 1 year	More than 1 year	
Routine infant^c,d,e^ (0 or 3 doses only)	2, 3, 4m	95.9	51/61	83.6	78 (52 to 89)	94 (80 to 98)	Overall 44 (-182 to 82) 12–23m 58 (-1,708 to 94) 24–35m 64 (-1,495 to 95) 36 + m 14 (-3,436 to 86)	P = 0.009
Routine 2 + 1 schedule^f^ (0 or 3 doses only)	2, 4, 12m	94.6	25/26	96.2	-43 (-5,759 to 77)	too few cases		NA
Infant catch-up (0 or 2 doses only)	5–11m	80.2	6/16	37.5	85 (55 to 96)	91 (-8 to 100)	83 (36 to 96)	P = 0.58
Toddlers catch-up (0 or 1 dose)	1–2y	80.2	11/27	40.7	83 (61 to 93)%	96 (87 to 99)	Overall 89 (81 to 94) 12–23m 92 (66 to 99) 24–47m 77 (11 to 95) 48–95m too few cases 96m + 75 (3 to 94)	P = 0.03
Pre-school catch-up (0 or 1 dose)	3–4y	71.9	7/47	14.9	93 (85 to 97)			
Infant school catch up (0 or 1 dose)	5–6y	57.7	3/23	13.0	89 (63 to 98)	95 (88 to 98)	Overall 94 (90 to 96) 12–23m 93 (80 to 98) 24–47m 87 (63 to 96) 48–95m 97 (87 to 100) 96m + 95 (76 to 99)	P = 0.33
Junior school catch up (0 or 1 dose)	7–10y	84.2	3/10	30.0	92 (65 to 99)			
Secondary school catch-up^g^ (0 or 1 dose)	11–16y	85.0	8/55	14.5	97 (94 to 99)			
Sixth form catch-up^d^ (0 or 1 dose)	17–18y	60.1	5/51	9.8	93 (82 to 98)			
Total	All ages	NA	119/316	37.7	NA	NA	NA	NA

CI: confidence interval; m: months; MCC: Meningococcal serogroup C conjugate; NA: not available; y: years.

^a^ Unvaccinated cases are based on the date of birth cohort each individual belongs to.

^b^ Trend analysis was done by logistic regression for the odds of vaccination in cases against log-time since vaccination with an offset of the log-odds of matched coverage. The exception was for the infant catch-up, where time was modelled as within a year and beyond a year as numbers were too small to model a trend.

^c^ Using DoH cover with THIN partial vaccination correction.

^d^ Including cases from January 2000.

^e^ Including one vaccinated case scheduled for three infant doses but only given a single dose after 12 months of age.

^f^ Including cases from April 2006.

^g^ Including cases from April 2000.

### Molecular characterisation

**Figure 2:**
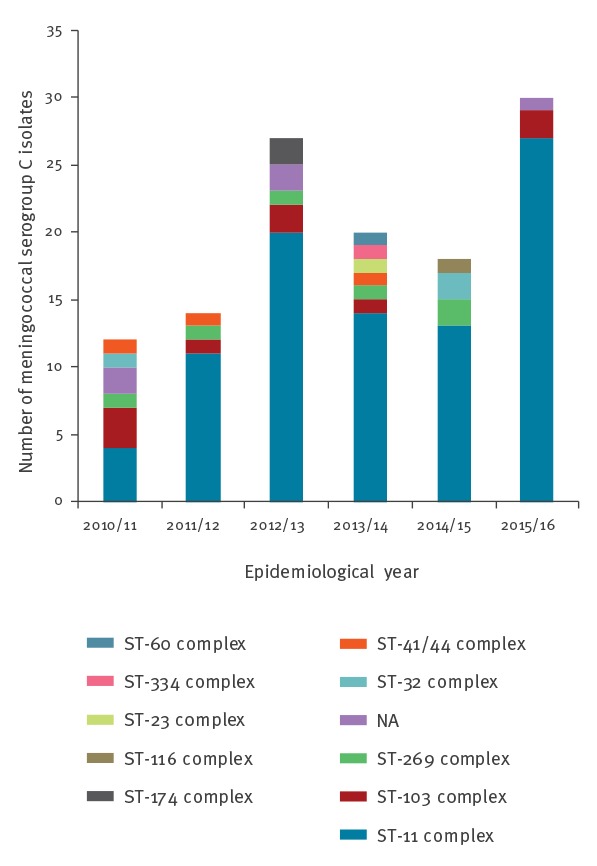
Clonal complex distribution among meningococcal serogroup C case isolates, England, 2010/11–2015/16

**Figure 3:**
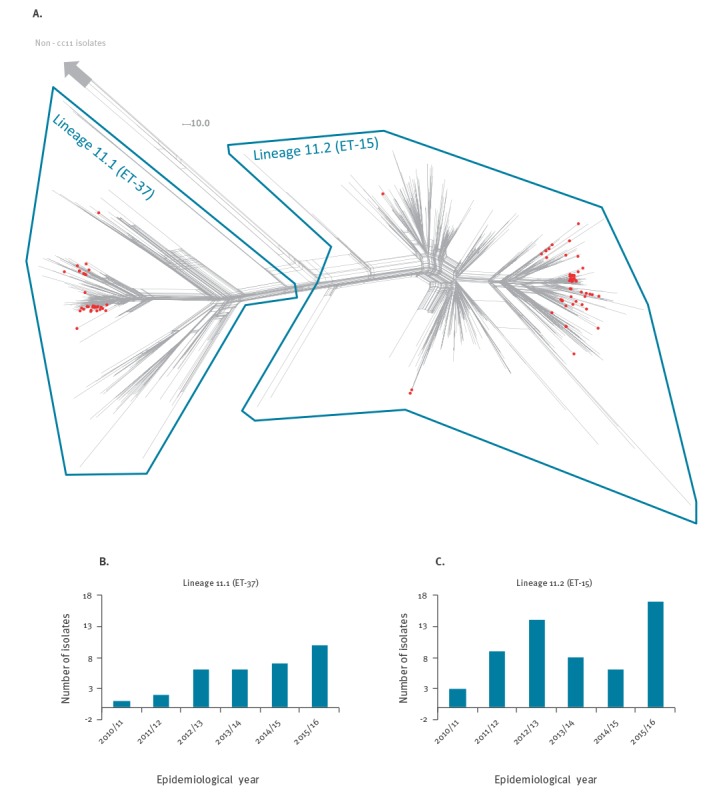
Diversity and temporal distribution of meningococcal serogroup C case isolates, England, 2010/11–2015/16

During 2010/11–2015/16, there were 182 laboratory-confirmed MenC cases in England’s national dataset, of which 122 were culture-confirmed. Molecular characterisation was available for 121 isolates, 89 of which (73.6%) belonged to the ST-11 clonal complex (cc11); ranging from 4 of 12 in 2010/11 to 27 of 30 in 2015/16. Other clonal complexes included cc103 (n = 9), cc269 (n = 6), cc-unassigned (n = 5), cc32 (n = 3), cc41/44 (n = 3), cc174 (n = 2) and a single isolate each for cc116, cc23, cc334 and cc60 (Figure 2). The population structure of cc11 (minus the MenW-associated lineage 11.1 sublineages) [18] comprised two main lineages, lineage 11.1 (corresponding to ET-37; n = 245; 6 MenB, 229 MenC, 1 MenY and 9 serogroup unspecified) and lineage 11.2 (corresponding to ET-15; n = 649; 77 MenB, 536 MenC, 3 MenW and 33 non-groupable/serogroup unspecified) (Figure 3a).

A) Neighbor-net phylogenetic network based on a comparison of 1,546 core genes among all available cc11 genomes on the PubMLST database, excluding predominantly-serogroup W lineage 11.1 sublineages (final n = 898; accessed 07/10/16). The English meningococcal serogroup C case isolates from 2010/11 to 2015/16 (red dots) were mainly distributed among several closely related lineage 11.1 and lineage 11.2 clusters, respectively. B) Cases due to lineage 11.1 isolates have increased since 2010/11, whilst C) cases due to lineage 11.2 isolates have fluctuated.

The English cc11 case isolates from 2010/11 to 2015/16 belonged to both lineage 11.1 (n = 32; 36.0%) and lineage 11.2 (n = 57; 64.0%), where they mainly belonged to several closely-related clusters (Figure 3a). The predominant PorA subtypes were P1.5,2 (93.8%) and P1.5–1,10–8 (96.5%), respectively. Since 2010/11, the number of lineage 11.1-related cases increased from one to 10 per year, while the lineage 11.2-related cases varied between three (2010/11) and 17 (2015/16) (Figure 3b,c). Isolates from vaccine-failure cases were randomly distributed among the overall English MenC cc11 population (not shown).

### Serosurvey

**Figure 4:**
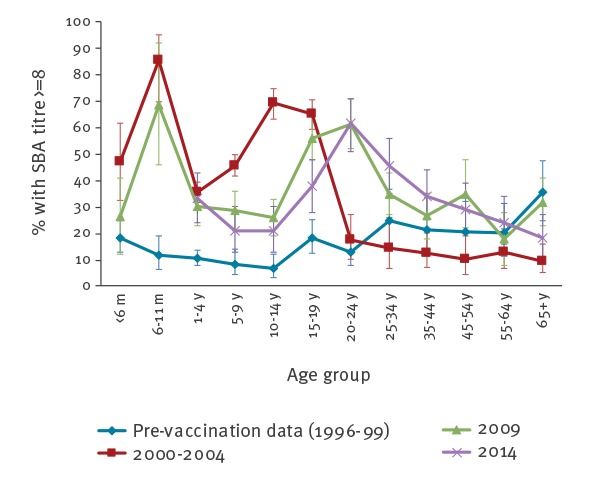
Seroprotection against serogroup C meningococci measured by proportions with serum bactericidal antibody (SBA) titres of ≥ 8

Results were available for 993 samples collected in 2014 and 323 (33%; 95% CI: 30.1 to 35.9) had SBA titres ≥ 8. The proportion of individuals achieving the seroprotective threshold was remarkably similar across the age groups in 2014 compared with 2009 (Figure 4). In contrast, much higher proportions of 5–19 year-olds were seroprotected during 2000–04, the period immediately after the 1999/2000 national catch-up for 0–18 year-olds. This cohort is now older and responsible for the secondary peak of seroprotection among 15–34 year-olds in subsequent serosurveys. Notably, only 38% of 15–19 year-olds achieved the seroprotective threshold in 2014 compared with 56% in 2009. Only 25% (75/299) children aged 1–14 years were seroprotected against MenC in 2014.

SBA: serum bactericidal antibody.

Comparison of levels in 2014 with the previous surveys conducted before MenC conjugate vaccine introduction in 1996–99 and following introduction in 2000–04 and 2009.

The 14/15 year-olds and 16 year-olds were separated in 2014 because they received different primary vaccination (Table 3) and to maintain consistency with the 2009 survey. Analysis by birth cohort and eligible vaccine schedule indicated higher GMT (10.6; 95% CI: 4 to 29 vs 3.7; 95% CI: 2 to 6) and proportions with SBA titres > 8 (28.2%; 95% CI: 15 to 44 vs 17.0%; 95% CI: 8 to 30) among 14–15 year-olds (the cohort eligible for the adolescent booster) in 2014 compared with 2009 (Table 3). In the younger age groups, GMTs and proportion protected were generally lower in 2014 compared with 2009 (Table 3).

**Table 3:** Proportion of samples with serum bactericidal antibody titres ≥ 8 and geometric mean titres by birth cohort and vaccination schedule

Vaccination schedule for birth cohort	Birth period	Age in 2009 (years)	Age in 2014 (years)	2009 Survey (95% CI)^a^			2014 Survey (95% CI)		
				n	% ≥ 8	GMT	n	% ≥ 8	GMT
2006 schedule: 2 + 1 schedule	2006–08	1-3	6–8	133	31.6 (24 to 40)	7 (5 to 10)	68	19.1 (11 to 30)	4.2 (3 to 6)
Three dose infant schedule	2004–05	4-5	9–10	63	30.2 (19 to 43)	5.7 (4 to 9)	37	24.3 (12 to 41)	3.5 (2 to 5)
	2001–03	6-8	11–13	76	29.0 (19 to 41)	4.8 (3 to 7)	52	21.2 (11 to 35)	3.5 (2 to 5)
	1999–2000	9-10	14–15^b^	53	17.0 (8 to 30)	3.7 (2 to 6)	39	28.2 (15 to 44)	10.6 (4 to 29)
Catch-up in second year of life	1998	11	16 ^b^	32	15.6 (5 to 33)	3.4 (2 to 6)	14	35.7 (13 to 65)	7.6 (2 to 28)
Toddler/preschool	1995–97	12-14	17–19^b^	101	31.7 (23 to 42)	7.2 (5 to 11)	54	38.9 (26 to 53)	8 (4 to 15)
Primary school	1990–94	15-19	20–24^b^	132	56.1 (47 to 65)	27.5 (18 to 43)	97	61.9 (52 to 72)	20.6 (12 to 35)
Secondary school	1982–89	20-27	25–32	165	55.8 (48 to 63)	28.3 (19 to 42)	54	44.4 (31 to 58)	15.80 (7 to 33)

CI: confidence interval; GMT: geometric mean titre.

^a^ Data from Ishola et al., 2009.

^b^ These age cohorts would include some individuals targeted for MenC vaccination as part of the adolescent/Fresher MenC vaccination introduced in 2013.

## Discussion

### Epidemiology

MenC disease in England remains at very low levels with an incidence of 0.08/100,000 in 2015/16, an overall reduction of 95.6% compared with 1998/99 before MCC vaccination was introduced. Nearly a third of cases are currently diagnosed in 25–44 year-old adults, many with clinical, social and travel-related risk factors. Overall case fatality remains unacceptably high. Updated VE estimates indicate a rapid decline in protection after 1 year following routine infant immunisation, but this remains high in children immunised at school age (5–18 year-olds) up to at least 8 years after vaccination.

The low MenC disease incidence is reassuring and is consistent with continued herd protection in the wider population and high direct protection in those who have been vaccinated. There have been no cases reported among at-risk individuals, currently defined as those with asplenia, splenic dysfunction or complement deficiency. Those born outside the UK (who are less likely to be immunised with MCC, even if eligible on entry), recently travelled and/or socially mixing with these populations accounted for a quarter of all cases, while a significant proportion of the other cases were living in shared or supported accommodation, which is a known risk factor for IMD [23]. An increased risk of IMD in HIV-positive individuals has recently been reported [24], even among those receiving appropriate antiretroviral therapy. Over the past 5 years, six HIV-positive individuals were identified among 160 MenC cases in England, all in the 25–44 year age group, including two who were non-UK born and had recently travelled abroad: Outbreaks of MenC disease in MSM populations have been reported in North America and Europe [25,26]. Whole genome sequencing previously showed that MenC case isolates among MSMs in England, while all belonging to lineage 11.2, were interspersed among sporadic community case isolates and did not form a discrete cluster [27].

### Molecular characterisation

Recent MenC cases in England were caused by diverse meningococcal strains, representing at least 10 different clonal complexes over the past 6 years. Most cases, however, were caused by two diversifying cc11 strains, one each from the two recently-resolved major cc11 lineages (lineages 11.1 and 11.2) [27,28]. Our findings warrant continued close monitoring, as lineage 11.1 cases have increased steadily over the past 6 years, while 2015/16 also saw the greatest number of lineage 11.2 cases. In addition to high case fatality rates for cc11 in general, the presence of lineage 11.2 isolates is concerning for several reasons. Evidence of frequent MenB to MenC capsule switching is coupled with poor potential coverage by, and the ability to escape from, several existing and experimental MenB vaccines [18]. Furthermore, distinct lineage 11.2 populations have independently acquired traits that may facilitate urogenital colonisation [29,30]. The acquisition of intact *aniA* alleles encoding nitrite reductase may enable survival in microanaerobic environments and has been associated with outbreaks of invasive disease among MSM in the US and Europe [30-32] and urethritis among heterosexual males in the US [29,30]. The urethritis-associated strain has also lost its ability to express a capsule, a further gonococcal trait that may facilitate adherence to the urogenital mucosa.

### Serosurvey

Seroprevalence studies have proven valuable in improving our understanding of population immunity, inform mathematical modelling and can complement disease surveillance to help inform national vaccine policy [8-10]. The 2014 serosurvey confirmed the rapid decline in immunity after infant and toddler immunisation (also observed with the 2009 serosurvey) as well as the low proportion of teenagers protected against MenC disease around the time when the adolescent programme was introduced. Similar trends have been reported in the Netherlands, where a large catch-up campaign targeting 1–19 year-olds in 2002 was followed by routine childhood immunisation at 14 months of age only. Here, a recent serosurvey found only 19% of 10 year-olds were protected 9 years after a single MCC dose at 14 months [33]. The successful control of MenC disease in the UK is attributed to the large catch-up campaign in 1999/2000. Teenagers and young adults have the highest nasopharyngeal meningococcal carriage rates and are considered the main source of spread. MCC vaccines not only protect against invasive disease but also against nasopharyngeal acquisition, thereby inducing herd protection across all age groups [4,6]. The current cohort of younger children with poor seroprotection is most likely protected against disease because of this indirect protection.

Most adolescents in 2014 will have received a three-dose infant MCC schedule without a booster. The low proportion of teenagers achieving protective antibody thresholds in the 2014 serosurvey indicated there was poor long-term direct protection in the cohort that was only immunised in infancy and ongoing herd protection across the population could be threatened. This observation, predicted after the 2009 serosurvey, helped support the inclusion of MCC in the adolescent programme in 2013 with the aim of providing high antibody concentrations throughout the peak years of carriage [7]. The 2014 serosurvey is already demonstrating higher MCC antibodies among 14–16 year-olds compared with the same age group in the previous serosurvey. The emergency adolescent MenACWY programme in 2015 was implemented to provide direct and indirect protection, not only against MenC but also against the three other serogroups; particularly the emergent hypervirulent MenW strain also belonging to cc11 that was responsible for a quarter of all IMD cases across the UK by 2014/15 [7].

### Implications for other countries

In Europe, MenC disease incidence is currently low (< 1/100,000) in countries with and without routine MCC vaccination [3]. Disease in infants remains rare and thus in countries with routine MCC immunisation most cases occur in adults, with a median age of 41 years compared with 22 in countries without MCC vaccination programmes. The recent increase in MenC cases in France highlights the importance of maintaining high vaccine coverage, especially in adolescents, in order to sustain long-term direct and indirect disease control [34]. A number of countries across Africa, Latin America and elsewhere are also experiencing high MenC disease activity [35,36]. Travellers to such countries, including residents visiting family and friends in their home countries, should be appropriately immunised, advised to avoid high-risk behaviours, remain vigilant for symptoms and signs of meningococcal disease and seek immediate medical help if concerned while abroad and when returning to their home countries.

### Strengths and limitations

The strength of this study is the consistently high quality of the national surveillance programme with high case ascertainment [12] and active follow-up of cases since 1998/99. This has allowed us to monitor VE in different cohorts over time. Our meningococcal surveillance is complemented with clinical trials, to assess the immunogenicity of different schedules, and serosurveys to monitor population susceptibility. Together, these results have allowed us to adapt the national immunisation programme to provide maximal long-term protection in the most cost-efficient manner. Using the same methodology allows us to monitor population effects over time. A limitation of the serosurvey, however, is that we have no information on the vaccination status of the serum donors or their clinical state. Nonetheless, the large numbers of samples tested, along with the consistently high vaccine coverage nationally has allowed us to analyse the results by birth-cohorts.

### Conclusions

We found evidence for rapid waning of immunity following routine infant immunisation, even with a 12-month booster, which was introduced in 2006. Currently, most toddlers (1-4 years) and older children (aged 5-13 years) in England are not seroprotected against MenC disease and are dependent on the indirect protection offered through reduced carriage in young people aged 17-24 years. It is, therefore, reassuring that there is still evidence of herd protection in adults 17 years after mass vaccination of children in 1999. Immunisation of teenagers appears to be key for continued population impact, but also for direct protection before their social behaviour leads to greater exposure, carriage and invasive disease. We have demonstrated high short-term effectiveness of MCC vaccines in infants and pre-school children and the long-term effectiveness of MCC vaccines given to school-aged children and adolescents. These findings have led to several revisions of the UK immunisation schedule to maximise direct and indirect protection. The recent change to emergency MenACWY vaccination for teenagers was brought in to provide more rapid population control for the national MenW outbreak. It should also provide continued direct and population control for MenC and help establish this for MenY disease.

Continued epidemiological and microbiological surveillance will be critical to monitor current programmes which now include newer sub-capsular protein-based vaccines against MenB disease in infants. MenC disease continues to be rare but detailed case-based follow-up has identified certain groups that remain at higher risk for MenC disease despite containment within the wider population, these groups may benefit from targeted vaccination. Stochastic meningococcal outbreaks globally need to be carefully monitored, as well as the recent MenC outbreaks among MSM. High-resolution characterisation is critical to monitor circulating strains and detect known and unknown emerging threats.
